# Arsenic Exposure in Infancy: Estimating the Contributions of Well Water and Human Milk

**DOI:** 10.1289/ehp.123-A137

**Published:** 2015-05-01

**Authors:** Charles W. Schmidt

**Affiliations:** Charles W. Schmidt, MS, an award-winning science writer from Portland, ME, has written for *Discover Magazine*, *Science*, and *Nature Medicine*.

Studies of mothers with high levels of arsenic exposure indicate the toxic element does not pass easily into human milk.^1^^,^^2^^,^^3^ In this issue of *EHP*, researchers test the hypothesis that breastfed infants have lower exposures than infants fed powdered formula mixed with private well water.^4^

Early life is a time of heightened vulnerability to arsenic, a naturally occurring element in bedrock and a widespread contaminant of groundwater linked to cancer, cardiovascular disease, and other adverse health effects.^5^^,^^6^ Municipal water in the United States is subject to a federal standard of 10 ppb for arsenic. But arsenic in private wells is unregulated, and homeowners are not required to test for it.

**Figure:**
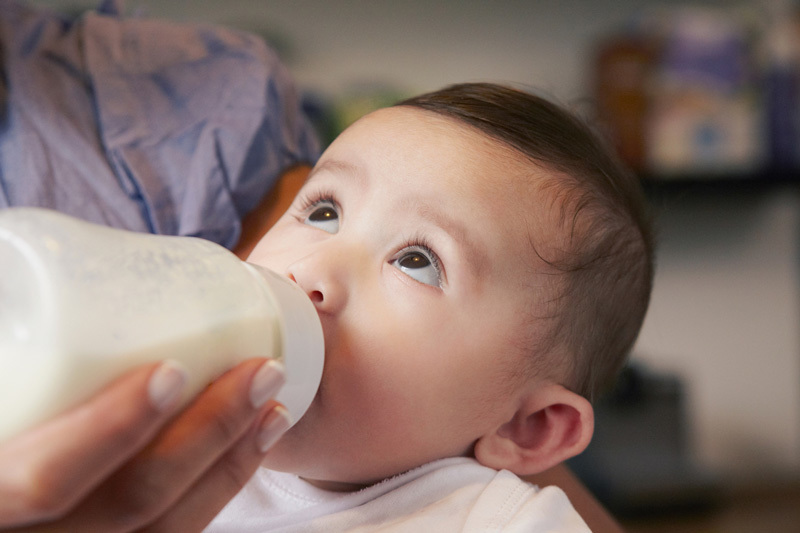
Parents who feed their babies powdered formula and make the formula with well water should test their water for arsenic. © Emma Kim/Image Source

“The take-home message is that if you’re feeding your baby powdered formula and making the formula with well water, then you should test your water for arsenic,” says Kathryn Cottingham, a professor at Dartmouth College and the study’s lead investigator.

These new findings add to mounting evidence that inorganic arsenic does not pass easily into breast milk. In a 2008 study conducted in Bangladesh, for instance, even women exposed to high levels of arsenic in groundwater had relatively little inorganic arsenic in their milk.^1^ That study also found that infants of women who reported exclusive breastfeeding had much lower arsenic levels in their urine at 3 months of age compared with 3-month-old infants who were not exclusively breastfed.

According to Cottingham, the current study set out to confirm these and similar findings in a cohort from the United States. The scientists worked with subjects enrolled in the New Hampshire Birth Cohort Study (NHBCS), which is investigating both maternal and child health effects of arsenic exposure during pregnancy. Launched in 2009, the NHBCS is directed by senior study author Margaret Karagas, principal investigator of the Children’s Environmental Health and Disease Prevention Research Center at Dartmouth.

Upon enrollment in the NHBCS, pregnant women had provided a sample of their home tap water for analysis. When their babies were approximately 6 weeks old, a subset of mothers filled out a 3-day food diary recording the type and amount of food consumed by the infant (infant formula and/or breast milk) along with the source and amount of water mixed with any powdered formula the baby received. The mothers also collected a sample of their babies’ urine on the third day. In addition, nine women provided a sample of their milk when their babies were 2–7 weeks old.

Cottingham emphasizes that the women had low levels of arsenic in their tap water, with just 1% exceeding 10 ppb. Still, urinary arsenic was detected in 97% of the infant urine samples. With a median of 0.17 µg/L, the median arsenic level in the urine of formula-fed infants was 7.5 times higher than the corresponding median detected in the urine of breastfed infants. Moreover, modeling results estimated that formula-fed infants had a daily arsenic intake that was 5.5 times that of breastfed infants.^4^

Lead author Courtney Carignan, a postdoctoral fellow now at the Harvard T.H. Chan School of Public Health, notes that breast milk samples also were low in arsenic. Five of the nine tested had detectable arsenic, with a median concentration of 0.31 µg/L.^4^

According to Cottingham, the researchers did not measure arsenic in the powdered formula consumed by the infants in this study. However, in a market survey published three years ago, NHBCS investigators found that arsenic in powdered formula—a mixture of dried cow’s milk, vegetable oils, lactose, vitamins, and other ingredients—was low.^7^ Cottingham and colleagues concluded that powdered formula and tap water probably contribute equally to arsenic exposures in formula-fed infants when tap water levels are low. As the amounts in tap water climb, she explains, they likely contribute a proportionately greater fraction of arsenic exposure in infants.

Ana Navas-Acien, an associate professor of environmental health sciences at the Johns Hopkins Bloomberg School of Public Health, says the new study reinforces a basic public health message: that homeowners who rely on private wells should have their water tested for arsenic, especially if they have infants and young children. “And there are many good reasons to breastfeed, and now protecting from arsenic provides one more,” she says. “The evidence suggests that arsenic exposure early in life can be detrimental later on, so protecting kids from these exposures is important.”
